# Mergers and acquisitions: does performance depend on managerial ability?

**DOI:** 10.1186/s13731-023-00296-x

**Published:** 2023-05-12

**Authors:** Diah Hari Suryaningrum, Abdul Aziz Abdul Rahman, Abdelrhman Meero, Pandu Adi Cakranegara

**Affiliations:** 1 Department of Accounting, Faculty of Economics and Business, Universitas Pembangunan Nasional Veteran Jawa Timur, Surabaya, Indonesia; 2 Department of Finance and Accounting, College of Business Administration, Kingdom University, Riffa, Bahrain; 3 Department of Management, Faculty of Business, President University, Cikarang, Indonesia

**Keywords:** Mergers and acquisitions, Managerial ability, Short- and long-term performance, Indonesia

## Abstract

Companies in difficult financial situations may seek to survive through mergers and acquisitions. Managers must be able to use company resources efficiently to maintain and improve competitiveness and sustainable advantages. Managers' ability to make strategic decisions may determine whether a merger and acquisition is successful. This study aims to reveal the role of the acquirer's managerial ability in mergers and acquisitions based on short- and long-term performance as well as the type of M&A. Two metrics are used to assess short- and long-term performance: the market-to-book ratio (MTBR) as an indicator of operating performance and the buy-and-hold abnormal return (BHAR) as an indicator of stock return performance. The research sample consists of 153 M&A cases conducted by companies registered with the Business Competition Supervisory Commission in Indonesia between 2010 and 2017, and the performance till 2020. We used regression and difference analysis to analyze the data. We find that managerial ability has a positive impact on MTBR operating and BHAR stock performance. This result confirms that the higher ability of the acquirer's manager will ensure a successful M&A in the long run. Investors and potential investors might consider managerial ability in choosing investments in companies after an M&A. This study contributes to the M&A literature by examining the role of MA in the short- and long-term performance of acquiring firms following M&As in Indonesia.

## Introduction

Business competition and environmental conditions cause companies to improve their performance through mergers and acquisitions (M&A). This is evidenced by the increasing number of mergers and acquisitions in Indonesia during the pandemic. Based on data from the Business Competition Supervisory Commission (KPPU), the Supreme Court's notification in 2019 and 2020 was 120 and 195 cases, respectively, indicating a 62.5% increase in M&As (KPPU, 2019, 2020). According to Sanny Iskandar, Chairman of the National Indonesian Employers Association (Apindo) for Property and Economic Zones, the difficult conditions caused by the coronavirus pandemic may encourage businesses to merge to survive. Meanwhile, acquisition transactions may be conducted within the context of acquisitions because the acquiree finds it difficult to survive in difficult circumstances (Julian, 2020).

The significant increase in mergers and acquisitions from 2019 to 2020 demonstrates that most business actors are extremely confident in conducting mergers and acquisitions due to their diverse motivations and goals. This means that mergers and acquisitions are still a viable strategic option for growing businesses. Furthermore, the presence of successful M&As can motivate and attract the attention of business actors to conduct M&As. Bank Mandiri, for example, was formed by the merger of the Indonesian Development Bank (Bapindo), Bank Dagang Negara, Bank Export–Import Indonesia, and Bumi Daya Bank. In Indonesia, it is still one of the largest banks today. Bank Mandiri is regarded by the public as an example of a banking sector that has successfully completed mergers and acquisitions (Wulandari, 2020).

However, some empirical studies contradict the positive results of company performance after M&As. Many companies fail to increase corporate performance after mergers and acquisitions, mainly when they are carried out during a pandemic that is clearly full of high uncertainty. One example occurred in the acquisition of So Good. PT Japfa Comfeed Indonesia (JPFA) is still vulnerable to being overshadowed by a decline in chicken demand amid the COVID-19 pandemic. JPFA's financial performance recorded a decline to Rp16.91 trillion (7%). Meanwhile, net profit fell into Rp155.11 billion (81%). The decrease in execution was brought about by frail interest, which caused the volume of animal feed and commercial farm day-old chicken (DOC) to decline (Julian, 2020). Research conducted by Borodin et al. (2020) proved that after deciding to conduct M&As, the financial performance of banks in Pakistan deteriorated. Many cases of failed mergers and acquisitions occurred. The findings of Wibowo and Hamdani's study (2020) indicate that corporate M&A activity has a negative effect on the generation of corporate value. The synergistic effect of M&A actions has also not been able to increase the value of the company and its shareholders. In a case study of M&A in the cement industry in Indonesia, the acquisition of state-owned cement holding Semen Indonesia over Holcim Indonesia in 2018 indicated that acquisition during excess capacity is a failure step taken by Semen Indonesia (Subiyanto, 2020).

Previous research indicates that the decision to pursue M&A does not always result in increased earnings and value for the acquirer's company after the transaction. The top management of the company is in charge of making investment decisions, funding, subsidizing, and other strategic decisions. The CEO, other senior executives, and members of the company's top management team all have a significant impact on business decisions and performance. Management literature has long recognized that differences in business performance can be explained by managers. Managerial ability is a necessary component of managerial characteristics (Salehi & Moghadam, 2019). According to Bertrand and Schoar (2003), managerial characteristics are a key success factor in economics, finance, and business practices. Managers must be able to use company resources to maintain and improve competitiveness and long-term advantages. Managers can effectively manage the company if they can efficiently control the company’s scarce resources, which will lead to a high level of efficiency (Chang et al., 2010). Furthermore, according to Cheung et al. (2017), managers with high abilities are more capable of improving the company's performance because they add value to the company.

Few studies have examined how managerial skills affect a company's performance (Chang et al., 2010; Cheung et al., 2017; Demerjian et al., 2012; Huang et al., 2014; Jiang et al., 2013; Mishra, 2014). Chang et al. (2010) asserted that a skilled manager can accurately predict environmental capital expenditures without significantly affecting spending in subsequent years. To obtain a higher rate of return and a higher market value, competent managers are better able to manage the business. An equipped chief officer can comprehend innovation and industry patterns, foresee item requests, put resources into projects with more significant yields, oversee representatives well, and recognize and better endeavor to exploit valuable opportunities (Demerjian et al., 2012; Mishra, 2014). In addition, Kallamu and Saat (2015) and Huang et al. (2014) assert that when earnings information is communicated to the market, managerial skill indicates that the market has a favorable reaction to the company's earnings information. However, there has never been a study that looked at the relationship between a company's short- and long-term performance in relation to M&As in Indonesia and managerial ability.

Due to the lack of research on this topic in Indonesia, this research looks into how long-term performance in mergers and acquisitions (M&A) relates to the acquirer's managerial ability (MA). A bibliographic study on M&A research in Indonesia indicates that there is no study about the influence of M&As on the concerned companies’ performance in short- and long-term periods. Most of the previous research on M&As sought to compare performance before and after M&As, whereas this research sought to demonstrate the influence of MA on company performance over short- and long-term periods. Additionally, since M&A is a strategic management decision, it is important to study the influence of MA on M&A companies’ performance in the short and long term. In line with resource-based theory, managers can achieve a firm's long-term competitive advantage by effectively and efficiently utilizing the company's resources (Barney, 1991, 1996). Cui and Chi-Moon Leung (2020) agree that empirical research on acquiring companies’ post-acquisition performance has not consistently uncovered evidence of improved performance. In addition, some business actors still lack insight regarding the influence of managerial ability (MA) in mergers and acquisitions (M&A). Whether managerial ability (MA) is an important factor in the success of mergers and acquisitions (M&A) or whether it will cause these businesses to fail, the purpose of this study is to investigate the connection between long-term performance in mergers and acquisitions (M&A) and the acquiring company's managerial ability (MA).

This research contributes to the literature and M&A practices in Indonesia by providing new evidence of the influence of M&As on the concerned companies’ performance. M&A action is a desirable strategy when a company faces an economic difficulty, such as in a pandemic situation. Since mergers and acquisitions are more than just a short-term trend because they sustainably affect company value, such strategies may have an impact on the company's sustainability. When choosing which businesses to invest in, both institutional and private investors are looking for a sustainable business (Prengel, 2021). Based on the M&A action from 2010 to 2017 and the company’s performance from 2011 to 2020, the results show that M&As affect companies’ operating performance (market-to-book ratio, or MTBR) in short- and long-term periods and stock returns (buy-and-hold abnormal return, or BHAR) in long-term periods. These results imply that investors should consider the managerial ability (MA) of the acquiring company regarding M&A transactions to maintain the sustainability of their investment.

The remainder of our paper is organized as follows: in the "Literature review and hypothesis" section, we review the related literature on M&As in Indonesian accounting standards, managerial ability, and company performance to develop our hypotheses. In the "Research method" section, we present the data, the sampling procedure, and our measurements of variables and research design. We report the results and discussion in the next section and conclude our study in the last section, which includes theoretical implications, managerial implications, limitations, and ideas for future research.

## Literature review and hypothesis

### Mergers and acquisitions in Indonesian accounting standard

M&As affect the competition between business actors in the relevant market and have an impact on consumers and the public. Such an impact may harm business competition through monopolistic practice or unfair business competition. Therefore, in Indonesia, the Business Competition Supervisory Commission (KPPU) will exercise control over mergers, consolidation, and/or takeovers to reduce the level of unfair competition in the Indonesian market, which may cause harm to society. KPPU was formed according to Government Regulation of the Republic Indonesia No. 57 of 2010 concerning M&A. Article 1 of the law defines a merger as a legal action taken by one or more businesses to merge with other existing businesses. The merger results in the transfer of the merging businesses' assets and liabilities to the business that accepts the M&A, and the merging businesses' status is terminated by law.

Based on the Guidelines for Indonesian Financial Accounting Standards (PSAK) No. 22 concerning business mergers, a business combination, commonly referred to as a merger, is the merger of two or more distinct businesses into a single economic entity. When two businesses merge, one company takes control of the other's assets and operations. While an acquisition is a business combination, the acquiring company gains significant control over the operations and assets of the acquired company by providing certain assets, recognizing a liability, or issuing shares (IAI, 2018). According to Sudarsanam (1999, p. 2), acquisition in business phrasing is characterized as the takeover of possession or return of offers or resources of an organization by another organization, and the organization being assumed control over still exists as per the arrangements of a different lawful substance. PSAK number 22, concerning business mergers, stated that control in acquisitions is assumed to be obtained if one of the merging companies acquires more than 50% of the voting rights, unless it can be proven that there is no control even though the ownership is more than 50%. Although one of the merging companies does not represent more than 50% of the voting rights in the other company, the acquiring company can still be identified if the shares of one of the merging companies are acquired (IAI, 2018).

Mergers and acquisitions are carried out by businesses for a variety of reasons, including to improve efficiency, expand operational synergies, or increase market share. This methodology can later be used to expand the organization's business. This study aims to determine whether managerial ability (MA) is related to the performance of a company because mergers or acquisitions are managerial decisions.

### The relationship between managerial ability and market-to-book ratio (MTBR)

Managerial ability is related to resource-based theory (Barney, 1996). According to this theory, a company's ability to maximize revenue is rationally dependent on the management skills of its managers. Companies that are able to compete will typically possess a variety of resources that can be utilized to achieve a long-term advantage over competitors. A sustainable competitive advantage can be achieved by utilizing resources from corporate strategy. Managers must comprehend the connection between resources, competitive advantage, and future revenue earnings before using the resource. Competent managers have managerial abilities that are believed to allow them to design appropriate strategies to optimize the use of company resources for generating sustainable competitiveness. Therefore, competent managers are able to manage input efficiently to produce optimal output. In addition, they are able to project future business conditions so that they can ensure that the company has good performance in the future.

However, in agency theory, the relationship between the agent (firm management) and the principal (boards and shareholders) is an agency relationship, where rational parties—the agent and the principal—will seek to maximize their individual utility (Jensen & Meckling, 1976). Since the decision to engage in an M&A depends on the CEO and/or executive directors, the board and shareholders will choose managers who can maximize the benefit of M&A actions and create a sustainable company after the M&A. However, the principal may not like the outcomes that are produced by inadequate monitoring, conflicting incentives, and incomplete and asymmetric information. When principals try to get agents to act in their own interests, incomplete and asymmetric information leads to agency issues. A global analysis of 7059 M&A actions executed from 2010 to 2017 using the Value Recognition Index (VRI) found that one of every three cases has a positive VRI (GPMIP, 2022). This means that M&A actions may create (increase) or destroy (decrease) a company's value.

According to Cui and Chi-Moon Leung (2020), the company's top management is responsible for investment decisions, financing, and other strategic decisions. The company's top management, which includes the executive directors, CEO, and other executives, also has an important role and becomes a significant factor in making company decisions. Therefore, the main point here is that managerial ability is the main factor in creating value, synergies, and both in the short-and-long term company performance. Controlling shareholders will use managers who can manage and design efficient business processes and make decisions that maximize profits and value to the company, especially for controlling shareholders. In addition to managing the company well, managers also have an obligation to provide transparent information related to the company's performance to stakeholders with an interest in the company in financial reports that are prepared every reporting period (Ng & Daromes, 2016). Therefore, this obligation may reduce agency problems and information asymmetry.

Rajan and Zingales (1995) defined the market-to-book ratio (MTBR) to determine the difference between the stock's market price and its book value. A comparison or ratio between book value per share and market value per share is known as the market-to-book ratio. The long-term performance of the company is measured in this study using the market-to-book (MTB) method. The essential methodology used to gauge long-haul stock returns in this study is purchase and hold. The BHAR (buy-and-hold abnormal return) strategy, according to Doeswijk et al. (2006), can be used to measure long-term stock performance calculated by (stock returns minus market returns) divided by the period.

According to Halim and Widjaja (2020), stock returns are stock income and are changes in the value of stock prices for period t with t-1. The greater the difference in stock prices, the higher the return on investment. In addition, stock returns can motivate investors to invest and provide views regarding rewards for investors' courage in taking risks in their investments. Tandelilin (2010, p. 105). The two main components of stock returns are as follows: gain and yield. Gain is an advantage that investors earn when the selling price is higher than the purchase price in the secondary market. The term "yield" refers to periodic income or cash flow, such as dividends or interest. The market-to-book (MTB) ratio can be used to determine book value, which can have an impact on dividend payments. The likelihood of an investment opportunity for the company tends to be greater the higher the MTB value Amidu and Abor (2006). Shareholder value can be directly measured by the share price, which can also reflect expectations regarding future performance (Cui & Chi-Moon Leung, 2020).

Mergers and acquisitions will attract investors to keep their investments or just want to invest in the company. The company's main objective in conducting mergers and acquisitions is to achieve synergies and improve the company's long-term operating performance for the sake of business continuity. Long-term operating performance can be measured in financial statements using financial ratio analysis, as in this study, which uses the market-to-book ratio (MTBR) to measure long-term operating performance as well as 1, 2, and 3 years after the M&A.

It is necessary to have good managerial skills in planning, managing, and being responsible for complex tasks, including accepting risks when the merger and acquisition decision is made, to achieve the synergy of mergers and acquisitions. The research results conducted by Cui and Chi-Moon Leung (2020) and Putri and Suryaningrum (2021) demonstrated that in mergers and acquisitions, M&As positively influence a company's long-term operating performance. According to resource-based theory (Barney, 1996), the resources used by managers to achieve competitive advantage require an understanding of the relationship between resources, managerial capabilities, competitive advantage, and the revenue that the company earns in the long run. The benefits of M&As will be readily obtained if the company has sufficient motivation to carry out M&As and then achieve the M&As objectives correctly and on target. In addition, the core of this theory is about using the company's strategic resources to build a long-term competitive advantage. Based on this explanation, the first hypothesis is as follows:

#### H1:

 The acquiring company's managerial ability positively affects the market-to-book ratio (MTBR).

The derived hypotheses are as follows:

#### H1a:

The acquiring company's managerial ability positively affects operating performance (MTBR) 1 year after mergers and acquisitions.

#### H1b:

The acquiring company's managerial ability positively affects operating performance (MTBR) 2 years after mergers and acquisitions.

#### H1c:

 The acquiring company's managerial ability positively affects operating performance (MTBR) 3 years after mergers and acquisitions.

The first hypotheses are tested both without and with the control variables.

### The relationship between managerial ability and buy–hold abnormal return (BHAR)

According to Demerjian et al. (2012), managerial ability is essential in merger and acquisition activities because managers are more capable. They are considered to know the company's macroeconomic conditions better when estimating costs such as bad debts and then more deeply about future benefits. They are expected to be obtained by the company and are more responsive to complex tasks within the company when the business combination is carried out.

As an alternative to evaluating long-term performance in mergers and acquisitions, there are market-based long-term performance measures such as stock returns in addition to the operating performance that is based on accounting measures. The goal of using stock returns as a measure of long-term performance in mergers and acquisitions is to create means and wealth for shareholders. The offer price is an immediate measure of investor esteem and can mirror the organization's drawn-out exhibition assumptions post-acquisition. The results of the research conducted by Doukas and Zhang (2020), Chen and Lin (2018), and Cui and Chi-Moon Leung (2020) proved that managerial ability positively influences post-acquisition stock returns. Based on these considerations, the second hypothesis is as follows:

#### H2:

The acquiring company's managerial ability positively affects buy-and-hold abnormal return (BHAR).

The derived hypotheses are as follows:

#### H2a:

The acquiring company's managerial ability positively affects stock returns one year after mergers and acquisitions.

#### H2b: 

The acquiring company's managerial ability positively affects stock returns two years after mergers and acquisitions.

#### H2c

The acquiring company's managerial ability positively affects stock returns three years after mergers and acquisitions.

The second hypothesis is tested both without and with the control variables.

### The difference of managerial ability and corporate performance in horizontal and diversified M&As

Redistribution theory is relevant for understanding horizontal and diversified M&A decisions. Ahern and Weston (2007) stated that redistribution theories explain five categories of M&As by stakeholder groups from a business combination, namely, tax gains for bidders, increasing market power, bondholders, workers and pension funds. Each of these categories is part of the motivation of business actors to carry out mergers and acquisitions. A horizontal and diversified strategy is a viable business development strategy through mergers and acquisitions. By connecting two or more businesses in the same industry, this horizontal merger aims to reduce competition or boost efficiency. The industry's market structure will become more concentrated as a result of this horizontal merger. The term diversified M&A refers to transactions that take place across industries.

When businesses participate in mergers and acquisitions, managers can use their abilities to identify and evaluate potential targets. Good managers can judge which horizontal acquisitions are better than diversified (cross-industry) acquisitions in achieving the objectives of mergers and acquisitions. According to Cui and Chi-Moon Leung (2020), in mergers and acquisitions, three main types of knowledge are required for managerial ability: generic or general, industry specific, and company specific.

When the target company and the acquirer are in the same industry, managers are better able to spot inefficiencies (Capron & Hulland, 1999). It is because the managers who manage the business already have experience in the line of business, they live in. In addition, when the companies were merged, managers of the acquiring company were also aware of the targeted company's key risks, economic performance, and economic drivers. This finding is in line with research conducted by Cui and Chi-Moon Leung (2020), which shows that managerial ability positively influences long-term post-acquisition performance when carried out in horizontal acquisitions rather than diversified acquisitions. In line with the description above, the third hypothesis is as follows:

#### H3:

 The acquirer's managerial ability in horizontal acquisitions on operating performance (MTBR) and stock returns (BHAR) is different from that in diversified acquisitions.

The derived hypotheses are as follows:

#### H3a: 

The acquirer's managerial ability in horizontal acquisitions on operating performance (MTBR) 1 year after M&As is different from diversified acquisitions.

#### H3b:

The acquirer's MA in horizontal acquisitions on operating performance (MTBR) 2 years after M&As is different from diversified acquisitions.

#### H3c:

 The acquirer's MA in horizontal acquisitions on operating performance (MTBR) 3 years after M&As is different from diversified acquisitions.

#### H3d:

The acquirer's MA in horizontal acquisitions on stock returns (BHAR) 1 year after M&As is different from diversified acquisitions.

#### H3e:

The acquirer's MA in horizontal acquisitions on stock returns (BHAR) 2 years after M&As is different from diversified acquisitions.

#### H3f:

The acquirer's MA in horizontal acquisitions on stock returns (BHAR) 3 years after M&As is different from diversified acquisitions.

## Research method

The M&A transactions data related to the studied cases have been collected from the published reports (2010–2017) by KPPU, which are aligned with the Indonesian government regulation for transparency and fair play in M&A. It requires a notification that must be submitted by business actors to the Commission if the merger has been legally effective and meets the requirements in accordance with the provisions of PP Number 57 of 2010. The population of the study consists of all public acquiring companies that conduct mergers and acquisitions and are on the Business Competition Supervisory Commission (KPPU) list for 2010–2017, which includes 412 cases. Table 1 shows the number of M&As per year. The number of uncommon acquisitions in the preceding long stretch of the example time frame arrived at its highest level of 90 in 2017. However, as the first pandemic crisis breaks out in 2020, M&A activity increases for the following year and reaches its peak. In contrast, the studies by Cui and Chi-Moon Leung (2020) and Ahn et al. (2010) show that M&A cases dropped due to the global financial crisis.

We obtained financial data and markets by accessing data from the Indonesia Stock Exchange (IDX) for validity and reliability. We used a purposive sampling technique in selecting the sample, with the following criteria: 1) The M&A transaction did not have an opinion from KPPU; 2) within three years after the M&A, the company does not engage in another M&A; 3) the acquirers’ companies are listed on the Indonesia Stock Exchange (IDX) for data validity and reliability; and 4) the company has complete information regarding variables in this study. Since this research examines long-term performance over 3 years, we need performance data for *t* + 1, *t* + 2, and *t* + 3. We use M&As from 2010 to 2017 and companies’ performance from 2011 to 2020. For example, in M&A transactions in 2017, companies' performance was analyzed for three years: *t* + 1 = 2018, *t* + 2 = 2019, and *t* + 3 = 2020. Based on purposive sampling, the sampled M&A includes 51 M&A cases and 153 (51 × 3) data observations (Table 1).

**Table 1:** Frequency of M&As from 2010 to 2017

Year	N	Percent	Cumulative percentage
2010	3	0.0073	0.0073
2011	43	0.1044	0.1117
2012	36	0.0874	0.1991
2013	69	0.1675	0.3666
2014	55	0.1335	0.5001
2015	51	0.1238	0.6239
2016	65	0.1577	0.7816
2017	90	0.2184	1.0000
Total	412	1.0000	

Data processed from the M&A announcement of the KPPU website (https://kppu.go.id/pemberitahuan-merger-year/). The M&A in 2018, 2019, and 2020 are 74, 120, and 195, respectively. The number of M&A transactions greatly increased in 2020 when the economy was down from the COVID-19 pandemic. This indicates that M&A action is a desirable strategic decision for companies to overcome crises and maintain their sustainable development

### Variable measurements

#### Independent variable

The independent variable is managerial ability. The managerial ability measurement follows the assessment by Demerjian et al. (2012). We use data output and input from financial statements. The efficiency of manager ability (MA score) is calculated using data envelopment analysis (DEA). The output is sales as a primary indication of the value of the company’s product. The inputs are the cost of goods sold (COGS), selling and administrative expense (SG&A), net property plant equipment (PPE), operating lease, research and development (R&D), goodwill, and other intangible assets.

#### Dependent variables

The dependent variables are the market-to-book ratio (MTBR) and the buy-and-hold abnormal return (BHAR) as proxies for company performance for short- and long-term periods, respectively. We measure the MTBR as the operating performance following Gitman (2009:169). By comparing the company's net assets to the market price of all outstanding shares, we determine whether a company's stock is overvalued or undervalued. The BHAR, as the stock return performance is measured, is derived from Doeswijk et al. (2006) and Abid and Muharam (2015). Because the decision to carry out an M&A transaction is strategic, in studies of financial and strategic management, stock returns have frequently been used to evaluate its performance.

#### Control variables

Following Cui and Chi-Moon Leung (2020), we use control variables of the size of the company, leverage, market–book ratio, tax loss, payment type (cash or stock), sales growth, and the type of M&A (horizontal and diversified M&A).

The variables' definitions and measurements are summarized in Table 2.

**Table 2:** Research variables and measurement

Variables	Definition	Measurement
Independent variable Managerial ability MA score	The value of managerial measures. The managerial ability can be seen in their efficiency of operational performance	Efficiency using DEA (Demerjian et al., 2012)
Dependent variables MTBR_i_	The market-to-book ratio of the acquiring firm after i-years of acquisition is adjusted for the industry median value of (*i* = 1,2,3 years)	$\mathrm{MTBR}i= \frac{\mathrm{Market capitailzation}}{\mathrm{Net book value}}$ (Gitman, 2009:169)
BHAR_it_	By subtracting the normal buy-and-hold return from the realized buy-and-hold return, the investor calculates abnormal returns by holding onto stocks for an extended period of time (*i* = year of M&A; *t* = 1,2,3 years)	Pit = share price of a company i 1 year after M&A Pi0 = share price of a company i at M&A D1 = Cumulative dividend of stock i 1 year after M&A It = IHSG 1 year after M&A I0 = IHSG at M&A (Fakhri, 2019)
Control variables		
SIZE	The company's acquired market value at the end of the fiscal year prior to the takeover announcement, expressed as a logarithmic value	$\mathrm{Size}=\mathrm{Ln total assets}$ (Cui & Chi-Moon Leung, 2020)
LEV	At the end of the fiscal year, prior to the acquirer's announcement of its takeover, the total assets' book value included both short-term and long-term company debt	(Cui & Chi-Moon Leung, 2020)
MB	The acquisition of the market value of the common stock to the book value of equity at the end of the fiscal year prior to the announcement of the takeover is used to calculate the market book ratio	$\mathrm{MB}=\frac{\mathrm{Market value}}{\mathrm{Book value}}$ (Cui & Chi-Moon Leung, 2020)
TAX-LOSS (TL)	Future tax losses adjusted for the acquirer's pre-acquisition market value	Tax losses/pre-acquisition market value (Cui & Chi-Moon Leung, 2020)
CASH	Whether the M&A is in cash or not	A dummy variable equals 1 if the transaction is only financed in cash and otherwise equal to 0 (Cui & Chi-Moon Leung, 2020)
STOCK	Whether the M&A is in share or not	A dummy variable equals 1 if the transaction is financed only by shares and 0 otherwise (Cui & Chi-Moon Leung, 2020)
SG	The current year's sales from the acquiring company are reduced by the previous year's sales, divided by the last year's assets, or it can be called sales growth	$\mathrm{SG}= \frac{\mathrm{Sales}(t)-\mathrm{sales}(t-1)}{\mathrm{Assets} (t-1)}$ (Cui & Chi-Moon Leung, 2020)
HORIZON	Whether the M&A is horizontal or diversified	A dummy variable is equal to 1 if the acquiring and target firms are in the same industry and 0 otherwise. (Cui & Chi-Moon Leung, 2020)

### Research design

The research design is an explanatory correlation research model based on a deterministic philosophy of cause and effect. According to deterministic philosophy, researchers can objectively reveal causal relationships (Bhattacherjee, 2012; Meressa, 2022). In addition, we use linear regression analysis to prove the hypothesis. The regression analysis test is believed to be better at predicting causal relationships and the influence of the independent and dependent variables (Chicco et al., 2021). We use a simple and multiple regression model or equation to test the influence of the managerial ability of the acquirer company on their post-M&A performance (H1 and H2) as follows:

1 $${\mathrm{Performance}}_{i}= \alpha + {\beta }_{1} {\mathrm{MA}}_{i}+ \varepsilon .$$

2 $${\mathrm{Performance}}_{i}= \alpha + {\beta }_{1} {\mathrm{MA}}_{i}+ {\beta }_{2} {\mathrm{SIZE}}_{i}+ {\beta }_{3} {\mathrm{LEV}}_{i}+ {\beta }_{4} {\mathrm{MB}}_{i}+ {\beta }_{5} {\mathrm{TL}}_{\mathrm{i}}+ {\beta }_{6} {\mathrm{CASH}}_{i}+ {\beta }_{7} {\mathrm{STOCK}}_{i}+ {\beta }_{8} {\mathrm{SG}}_{i}+ {\beta }_{9} {\mathrm{Horizon}}_{i}+ \varepsilon .$$

For the third hypothesis, we test the cross-sectional reaction of the subgroup difference between horizontal and diversified acquisition using the *t*-independent test.The regression model, *F* test, and *t* test are used to test hypotheses H1 and H2. In the *t* test, the variable can be said to have an effect on the criteria:

- If the Sig. value < 0.05, then the independent variable (*X*) has a significant effect on the dependent variable (*Y*).
- If the Sig. value > 0.05, then the independent variable (*X*) has no significant effect on the dependent variable (*Y*).

The direction of the influence depends on the value of the *t*-_count_. If the *t*-_count_ is positive, the influence is positive. If the *t*-_count_ is negative, the influence is in the opposite direction.

## Results and discussion

### Descriptive statistics

The main dependent, independent, and control variables are summarized in Table 3. The MTBR summary statistics for the acquirers' operating performance one, two, and three years after the acquisition are displayed in Panel A. The acquirers' long-term BHARs are summarized in Panel B for the 1-, 2-, and 3-year windows following the acquisition, respectively. The descriptive statistics show that the mean/median value of post-acquisition operating performance (or long-term returns) is partially negative, which is consistent with the results of previous studies. Demerjian et al. (2012) show that stock prices have negative reactions over one to three years after acquisitions. The value of our primary independent variable, MA_SCORE, is reported in Panel C. The descriptive statistics of the control variables are presented in Panel D of Table 2.

**Table 3:** Descriptive statistics

	MIN	Q1	MEDIAN	Q2	MAX	MEAN	STD
Panel A: operating. performance							
MTBR1	− 0.7848	0.0352	0.0819	0.3575	0.5078	0.1198	0.3128
MTBR2	− 0.8252	0.0308	0.0798	0.3022	0.4242	0.0916	0.2992
MTBR3	− 0.5384	0.0238	0.0782	0.2602	0.4179	0.1007	0.2273
Panel B: stock return performance							
BHAR1	− 0.6846	− 0.0975	1.1745	3.8271	13.2167	2.5973	4.0318
BHAR2	− 1.1317	− 0.1854	0.7573	4.0977	13.4133	2.4749	4.2176
BHAR3	− 1.9648	0.0029	0.7713	3.8715	13.3672	2.4334	4.2180
Panel C: managerial ability							
MA	0.1753	0.6729	0.8772	0.9277	1.1666	0.7921	0.2796
Panel D: control variables							
SIZE	28.5827	28.8597	29.5516	30.2527	32.1190	29.8203	1.1804
LEV	0.2125	0.4355	0.5173	0.6109	0.9125	0.5158	0.1822
MB	0.0131	0.0709	0.1072	0.4181	0.7201	0.2392	0.2498
TL	0.0000	0.0054	0.0416	0.1195	0.6145	0.1135	0.1800
CASH	0.0000	1.0000	1.0000	1.0000	1.0000	0.7857	0.4260
STOCK	0.0000	0.0000	0.0000	0.0000	1.0000	0.2143	0.4258
SG	0.0059	0.0701	0.1808	0.3271	0.8971	0.2336	0.2345
HOR	0.0000	0.0000	1.0000	1.0000	1.0000	0.7143	0.4690

Data processed from annual report and market information of the IDX (https://www.idx.co.id/) and company website. The fundamental descriptive statistics of the main variables that are associated with post-acquisition performance, managerial ability, and other control variables are presented in this table

### The relationship between managerial ability and market-to-book ratio (MTBR)

The F test to examine the goodness of fit of the regression model indicates that, without control variables, the MTBR operating performance for one, two, and three years is influenced by managerial ability. The significant values are 0.034, 0.028, and 0.044, lower than 0.05, while the regression model with control variables has a significant value of 0.162, 0.227, and 0.117, respectively, higher than 0.05. This means that managerial ability in the presence of the control variables does not affect the operating performance of MTBR (see Table 4).

**Table 4:** *F* test and *R*^2^ for regression model MTBR Source: SPSS, data processed (2021)

	Hypothesis	*F*	Sig	*R*^2^	Description
H1a	Regression without control variable (*Y* = MTBR1)	5.702	0.034	0.801	Strong relation
	Regression with control variable (*Y* = MTBR1)	2.522	0.162	0.322	Weak relation
H1b	Regression without control variable (*Y* = MTBR2)	6.277	0.028	0.764	Strong relation
	Regression with control variable (*Y* = MTBR2)	2.024	0.227	0.343	Weak relation
H1c	Regression without control variable (*Y* = MTBR3)	5.064	0.044	0.830	Strong relation
	Regression with control variable (*Y* = MTBR3)	3.058	0.117	0.297	Weak relation

The coefficient of determination, signified by *R*^2^ (Table 4), is a general proportion of decency of attack of the assessed relapse line (or plane, if more than one regressor is involved); that is, it gives the extent or level of the absolute variety in the dependent variable *Y* that is made sense of by all the regressors. *R*^2^ lies between 0 and 1; the closer it is to 1, the better the fit, and the closer it is to 0, the worse the fit. The coefficient of determination is used to compare the regression model between those without and those with control variables (Gujarati, 2015, p. 13–17). In Table 2, for H1a without control variables, the *R*^2^ is 0.801 (strong relation) greater than for H1a with control variables (*R*^2^ = 0.322 or weak relation). This means that the goodness of fit of the regression line of H1a without control variables is better than that with control variables. Hypothesis 1b (H1b) and H1c for long-term performance have the same meaning as *R*^2^. For the hypothesis test using a *t* test, it is recommended to use regression without the control variables.

Table 4 shows that the results of the coefficient of determination in regression without the control variables for MTBR1 (one year), MTBR2 (two years), and MTBR3 (three years) are 0.801, 0.764, and 0.830, respectively. This means that 80.1%, 76.4%, and 83% of managerial ability influenced operating performance, with the rest (19.9%, 23.6%, and 17%) influenced by other factors. The relation between MA and MTBR operation performance with control variables is weak for 1, 2, and 3 years after the MA. In contrast, the coefficient of determination with control variables indicates a strong relationship with *R*^2^ for MTBR1, MTBR2, and MTBR3, which are 0.322, 0.343, and 0.297, respectively.

Table 5 indicates that the significant values of the regression without control variables are 0.034, 0.028, and 0.044 (lower than 0.05). This value proved that managerial ability (MA) influenced corporate operating performance (MTBR) for one, two, and three years after the merger and acquisition. The β-coefficients and *t* values are all positive, which means that the relation between MTBR and managerial ability is in the same direction.

**Table 5:** *T* test of hypothesis without control variables Source: SPSS, data processed (2021)

Variables	MTBR1			MTBR2			MTBR3		
	*β*	*t* value	Sig.	*β*	*t* value	Sig.	*β*	*t* value	Sig
Constant	− 0.400			− 0.422			− 0.262		
MA	0.656	2.388	**0.034**	0.648	2.505	**0.028**	0.458	2.250	**0.044**

Te bold values indicate that the value is less than 0.05

As Table 6 shows, the results of the *t* test in the regression with control variables have a significant value greater than the specified significance level of 0.05. This means that MA, sales growth, market book ratio, leverage, stocks, tax losses, company size, and horizontal acquisitions do not affect operating performance 1, 2, and 3 years after the M&As.

**Table 6:** *T* test of hypothesis with control variables Source: SPSS, data processed (2021)

Variables	MTBR1			MTBR2			MTBR3		
	*β*	*t* value	Sig.	*β*	*t* value	Sig.	*β*	*t* value	Sig.
Constant	− 2.995			− 2.826			− 1.937		
MA	0.801	1.979	0.105	0.803	1.902	0.116	0.557	2.050	0.096
Size	0.061	0.765	0.479	0.057	0.683	0.525	0.038	0.709	0.510
LEV	0.421	0.758	0.483	0.358	0.618	0.564	0.282	0.756	0.483
MB	0.294	0.588	0.582	0.330	0.633	0.555	0.295	0.877	0.421
TL	− 0.556	− 0.987	0.369	− 0.497	− 0.846	0.436	− 0.392	− 1.038	0.347
Stock	− 0.249	− 0.906	0.406	− 0.295	− 1.031	0.350	− 0.191	− 1.034	0.349
SG	1.062	1.608	0.169	0.958	1.391	0.223	0.729	1.644	0.161
HOR	0.349	1.435	0.211	0.317	1.252	0.266	0.233	1.428	0.213

The results of this study without the control variables indicate that managerial ability positively affects operating performance (MTBR) one year, two years, and three years after mergers and acquisitions. This means that if the acquirer's managerial ability increases, it will be followed by an increase in operating performance (MTBR) 1–3 years after the merger and acquisition. This is in line with the opinion of Harahap (2010, p. 311) that the higher the value of the market-to-book ratio, the better the investor's assessment of the company's operating performance. The positive effect of the relationship between MA and operating performance means that the higher the MA, the more capable the manager is of managing resources to generate future earnings. As mentioned in resource-based theory (Barney, 1996), managers who have the high capability to manage a company’s resources will use the resources efficiently to create optimal income.

According to Ahern and Weston (2007), the test results support the redistribution theory that a merger or acquisition can be more productive, including generating profits by utilizing the company's assets. Mergers increase value, which makes the combining company add capabilities more quickly than through internal programs. When the economic environment is in crisis, there are more pressures on people to change and adapt quickly. As a result, MA plays a larger role in M&A transactions in environments that are changing at a faster rate (Ahern & Weston, 2007). The idea of modern finance supports it. Management decisions aim to increase shareholder wealth and increase firm value (Sudarsanam, 1999). In addition, the results of this study support previous results, which state that managerial ability has a positive effect on operating performance (MTBR) one year, two years, and three years after M&As (Cui & Chi-Moon Leung, 2020). It also supports the results of Chen and Lin (2018), who state that high MA will result in a high increase in profit.

Unfortunately, the results of the *t* test in regression with control variables indicated that the significance value for MTBR operating performance for one year, two years, and three years is not relevant to managerial ability. It means that MA, accompanied by leverage value, company size, market-to-book ratio, ability to bear tax losses in the future, control the type of company industry, and type of transaction, do not affect the operating performance one to two years after the merger or acquisition. The results of this study are contrary to the research of Cui and Chi-Moon Leung, (2020). This study shows that company size, leverage, market-to-book ratio, tax losses, shares, sales growth, and horizontal acquisitions also affect operating performance (MTBR) one and two years after the merger or acquisition. However, this study supports previous research by Wibowo and Hamdani (2020), according to which corporate mergers and acquisitions do not have a positive impact on the creation of corporate value and shareholder value in either the short or long term.

According to agency theory (Jensen & Meckling, 1976), the relationship between the principal, or shareholders, and the agents, or managers, is one in which shareholders give managers the authority to make the best decisions for the company. However, this relationship can be contradictory because managers may have their own interests that are different from those of shareholders. It can cause agency conflicts that trigger the necessary costs. Thus, the managerial focus can be divided between the problem and achieving common goals. Agency conflict showed that the manager does not always work according to the principal's plans and will trigger agency costs for the supervision of managers. This study found no effect of firm size, leverage, tax loss, market-to-book ratio, sales growth, stock, and horizontal acquisition on managerial ability. It can be interpreted by the existence of conflicts within the company that do not affect operating performance (MTBR) 1–3 years after M&A.

### The relationship between managerial ability and buy–hold abnormal return (BHAR)

The *F* test used to examine the goodness of fit of the regression model indicates that without the control variables, the BHAR's operating performance for one, two, and three years is not influenced by managerial ability. The significant values are 0.391, 0.415, and 0.444, respectively, higher than 0.05. At the same time, the regression model with control variables has significant values of 0.012, 0.005, and 0.002 lower than 0.05. This means that managerial ability with control variables affects the operating performance of BHAR for one, two, and three years after the merger and acquisition (see Table 7).

**Table 7:** *F* test and *R*^2^ for regression model BHAR Source: SPSS, data processed (2021)

	Hypothesis	F	Sig	R^2^	Description
H2a	Regression without control variable (*Y* = BHAR1)	0.792	0.391	0.062	No relation
	Regression with control variable (*Y* = BHAR1)	9.394	0.012	0.938	Strong relation
H2b	Regression without control variable (*Y* = BHAR2)	0.713	0.415	0.056	No relation
	Regression with control variable (*Y* = BHAR2)	13.772	0.005	0.957	Strong relation
H2c	Regression without control variable (*Y* = BHAR3)	0.627	0.444	0.050	No relation
	Regression with control variable (*Y* = BHAR3)	21.024	0.002	0.971	Strong relation

In Table 7, for H2a with control variables, the R^2^ is 0.938 (a strong relationship) and is greater than H2a without control variables (*R*^2^ = 0.062, or no relation). This means that the goodness of fit of the regression line of H2a with control variables is better than that without control variables. For H2b and H2c for long-term performance, the meaning of *R*^2^ is the same. For the hypothesis test using the *t* test, it is recommended to use regression with the control variables.

Table 7 shows that the results of the coefficient of determination in regression without the control variables for BHAR1 (one year), BHAR2 (two years), and BHAR3 (three years) are 0.062, 0.056, and 0.050, respectively. This means that only 6.2%, 5.6%, and 5% of managerial ability influenced operating performance, with the rest (93.8%, 94.4%, and 95%) influenced by other factors. The relation between Managerial Ability and BHAR operation performance with control variables is strong for 1, 2, and 3 years after the MA. The coefficient of determination with control variables indicates a strong relationship with *R*^2^ for BHAR1, BHAR2, and BHAR3, which are 0.938, 0.957, and 0.971, respectively.

Table 8 indicates that the significant values of the regression without control variables are 0.391, 0.415, and 0.444 (higher than 0.05). This value proved that MA had no influence on corporate BHAR stock performance for one, two, and three years after the M&As. The regression analysis results without control variables show that MA does not affect stock returns (BHAR) 1 year, 2 years, and 3 years after mergers and acquisitions, although it has a positive influence. The results of this study do not support the results of research conducted by Cui and Chi-Moon Leung (2020), according to which MA has a positive effect on stock returns (BHAR) 1 year after M&As.

**Table 8:** *T* test of hypothesis without control variables Source: SPSS, data processed (2021)

Variables	BHAR 1			BHAR 2			BHAR 3		
	β	t value	Sig	β	t value	Sig	β	t value	Sig
Constant	− 0.339			− 0.449			− 0.318		
MA	3.707	0.890	0.391	3.691	0.844	0.415	3.474	0.792	0.444

It is clear from Table 9, the results of the *t* test in the regression with control variables for one year after mergers and acquisitions, that managerial ability does not influence the operating performance of stock value (a *p* value of 0.098 is higher than 0.05). In contrast, managerial ability significantly affects BHAR stock performance for two and three years after the merger and acquisition (*p* values of 0.049 and 0.020 lower than 0.05).

**Table 9:** *T* test of hypothesis with control variables Source: SPSS, data processed (2021)

Variables	BHAR1			BHAR2			BHAR3		
	*β*	*t* value	Sig.	*β*	*t* value	Sig.	*β*	*t* value	Sig.
Constant	− 96.862			− 102.042			− 96.422		
MA	− 5.946	− 2.033	0.098	− 6.559	− 2.569	**0.049**	− 7.036	− 3.379	**0.020**
Size	3.459	6.026	**0.002**	3.647	7.280	**0.001**	3.477	8.512	**0.000**
LEV	− 10.747	− 2.676	**0.044**	− 11.703	− 3.340	**0.021**	− 11.572	− 4.050	**0.010**
MB	1.656	0.458	0.666	1.783	0.565	0.596	0.555	0.216	0.838
TL	12.433	3.057	**0.028**	13.621	3.838	**0.012**	15.405	5.323	**0.003**
Stock	3.135	1.579	0.175	3.349	1.934	0.111	4.091	2.896	**0.034**
SG	8.013	1.679	0.154	8.268	1.986	0.104	7.455	2.196	0.080
HOR	3.093	1.762	0.138	3.341	2.181	0.081	3.094	2.476	0.056

Te bold values indicate that the value is less than 0.05

The fact that MA did not influence BHAR for the first year may indicate that in M&A transactions, there are differences in culture between acquiring and acquired companies, so the management of the acquiring company still evolves and needs time to adjust to the transition. Company culture is defined as the beliefs and behaviors—a shared set of values, goals, attitudes, and practices—that determine the employee and management interaction in handling an organization (Tarver, 2021). Engert et al. (2019) see organizational culture as an unseen force that drives M&As. In the first year of M&A, there might be an agency problem that creates asymmetry in information since the company still needs time for management and employee transformation. The acquiring company manager needs to understand the acquired company's culture and proactively manage it. This process requires a comprehensive approach and may take more than one year. However, the MA affects BHAR in the long-term performance (2–3 years). According to agency theory, agency problems might not arise when managers and shareholders have the same opinion that creating company value will also create benefits for shareholders and managers' bottom lines. Both actors will maximize their utility.

Company size and tax losses for 1-, 2-, and 3-year performance are the control variables that positively affect performance. The *p* values for company size are 0.002, 0.001, and 0.000 < 0.05, and for tax losses, they are 0.028, 0.012, and 0.003 < 0.05. Leverage has a negative relation with BHAR performance for one, two, and three years (*p* values are 0.044, 0.021, and 0.010 < 0.05). The merger and acquisition using stock only influenced BHAR stock performance for three years (*p* value of 0.034 < 0.05). The reasoning behind the impact of company size on BHAR is that larger targets will be purchased by larger acquirers. The acquirer is more likely to pay more for a larger target that is said to be more established, more efficient, and, as a result, less risky. Companies that acquire such a target tend to be larger and, as a result, better able to manage value creation through M&As (GPMIP, 2022).

The regression analysis results with control variables indicate that firm size and tax losses have a positive effect. In contrast, leverage harms stock returns (BHAR) 1 year, 2 years, and 3 years after mergers and acquisitions. This means that the larger the company and the level of the manager's ability to bear tax losses in the future, the higher the return on shares generated one year after mergers and acquisitions. In addition, leverage with a negative influence direction means that if the leverage increases, the stock return value 1–3 years after the merger and acquisition will decrease. On the other hand, if leverage decreases, the stock value 1–3 years after the merger and acquisition will increase.

These results support Doukas and Zhang's (2020) research that if the manager's ability is lower, it will show a negative or low long-term stock return value. However, the results of this study do not support previous research conducted by Cui and Chi-Moon Leung (2020). They found that managerial ability, leverage, market-to-book ratio, stocks, sales growth, and horizontal acquisitions have a positive effect on stock returns 1–3 years after mergers and acquisitions.

Managers are given the authority to make the best decisions in accordance with agency theory, which explains the relationship between shareholders or owners and agents. They contradict each other, causing agency conflicts, as explained by Jensen and Meckling (1976), who state that managers do not always work under the principal's goals. The test with control variables also shows no effect on managerial ability, leverage, market-to-book ratio, stocks, sales growth, or horizontal acquisitions. This means conflict within the company do not affect stock returns one year after mergers and acquisitions. In addition, many other factors play a more critical role in influencing stock returns one year after M&As. However, this study does not support the results of research conducted by Doukas and Zhang (2020). Their study found that the payment method using stock for M&A transactions affects stock returns both in the short- and long-term periods.

The test on stock returns with control variables proves that managerial ability, firm size, leverage, tax losses, and stock transactions have a significant value less than 0.05. This indicates that the variables of firm size, tax losses, and stock positively affect BHAR stock returns 2 and 3 years after mergers and acquisitions. Suppose the size of the company is significant. In that case, the manager's ability to handle future tax losses is high, and the transaction control used in mergers and acquisitions in the form of shares will be followed by an increase in stock returns 2–3 years after the merger and acquisition are carried out. The results of this study support the opinion of Doukas and Zhang (2020) that the payment method used when shares are used as an M&A payment method can affect stock returns both in the short and long term. In contrast, the study results showed that managerial ability and leverage influenced BHAR stock returns 2–3 years after mergers and acquisitions, but negatively. This means that if MA and leverage increase, the value of stock returns 2–3 years after mergers and acquisitions have decreased, and vice versa.

Based on Table 9, for BHAR stock performance over 3 years, sales growth, the market book ratio, and horizontal acquisition variables have no effect on stock returns after mergers and acquisitions. The results do not support the results of previous research conducted by Cui and Chi-Moon Leung (2020). Managerial ability, sales growth, leverage, market book ratio, and horizontal acquisitions all positively affect long-term stock returns three years after M&As. The ineffectiveness of managerial ability, sales growth, leverage, market-to-book ratio, and horizontal acquisitions can be caused by factors described in agency theory related to agency conflict. According to Jensen and Meckling (1976), managers do not always follow the goals. Therefore, even though managers have the capability to manage the company’s resources, they might tend to fulfill their own interests rather than focus on the long-term goals of the company.

### The difference of managerial ability and corporate performance in M&A horizontal and diversified

Based on Table 10, the independent *t* test results indicate that all variables have a significant value greater than the set level of 0.05. It can be concluded that H0 is accepted, and Ha is rejected, meaning that there is no difference between MA, MTBR, and BHAR corporate performance in M&A horizontal and diversified.

**Table 10:** Independent test results Source: SPSS, data processed (2021)

Hypotheses		Levene's test Sig.	Sig.	Prob.
H3a	MTBR1	0.772	0.793	> 0.05
H3b	MTBR2	0.725	0.772	> 0.05
H3c	MTBR3	0.909	0.804	> 0.05
H3d	BHAR1	0.099	0.500	> 0.05
H3e	BHAR2	0.111	0.533	> 0.05
H3f	BHAR3	0.112	0.526	> 0.05

There is no effect of horizontal or diversified acquisition on MTBR operating performance or BHAR stock returns 1–3 years after M&A. This result is not supported by redistribution theory. According to Dharmasetya and Sulaimin (2009), companies that prefer to conduct mergers and acquisitions with cross-industry types will maintain better income stability, develop company value, and support companies' business activities and operations to secure their competitive position. When investors analyze the company's annual financial statements to consider investing in or extending investment in the company if it is associated with redistribution theory, diversified acquisitions have more opportunities for long-term stock returns. Thus, diversified acquisitions can be a company's motivation to increase company synergy.

## Conclusion

Based on the results, if the acquirer is able to maximize the benefits of synergies and minimize the costs of M&A activities involving strategic decisions made by the management of the acquiring company, then a merger and acquisition (M&A) is more likely to succeed. The empirical studies in Indonesia on acquiring companies’ performance after M&As indicate no difference in performance before and after M&As. However, more companies are involved in M&As to make strategic decisions in maintaining and sustaining a stable version, as indicated by the increase in M&As in 2019 and 2020. Using the market-to-book ratio of operating performance and the buy–hold abnormal return of stock as proxies for acquiring companies' performance. This study proves that managerial ability influences MTBR and BHAR differently.

Managerial ability influences MTBR operating performance without the existence of control variables such as firm size, leverage, tax losses, stock transactions, sales growth, and type of M&A. The influence is profound in the short and long performances. While there is no evidence that BHAR performance is influenced by managerial ability in the short-term horizon after the M&A, BHAR stock return is affected by managerial ability with the influence of control variables such as firm size, leverage, and tax losses only in the 2–3 years after the M&A. Under the crisis, the economic climate intensifies the need for swift change and adaptation. A company can change and adapt more quickly through M&As than through internal growth. As a result, MA plays a larger role in M&A transactions in environments that are changing at a faster rate.

The test of differences between the horizontal and diversified types of M&As is not proven. Both operating performance and stock returns are the same for horizontal and diversified M&As, whether in the short- or long-term, returns are the same for horizontal and diversified M&As, whether in the short or long term. This means that whether a company decides to take M&A action is not influenced by the type of M&A, since horizontal and diversified M&As have the same results in this study.

### Theoretical implications

Despite the large number of studies that have been documented on M&A action, surprisingly, there are a relatively small number of studies carried out to examine M&A action that focus on the role of MA on post-company performance for short- and long-term periods. Therefore, this paper contributes to filling the gaps in the literature on strategic management and identifies a resource-based approach to managerial abilities. Previous research tends to use nonfinancial measurement for MA rather than financial measurement. This research adds to the few financial measurements of MA in Indonesia. In addition, previous research has focused more on the capital markets of developed countries than on those of developing countries such as Indonesia.

### Managerial implications

According to these findings, managerial ability (MA) determines whether M&A as a strategy to maintain a company's performance succeeds or fails. The process and result of M&As involve many variables that may not have yet been researched. This research provides some insight into the role of managers and their ability to manage M&As, especially in Indonesia. Using the operating and long-term stock return performances, this study indicates that MA affects these performances. Therefore, it is essential for investors and potential investors to know more about the management team involved in M&A transactions. Managers who make the strategic decision to take M&A action, must consider the long-term performance of the company to maintain its sustainability after the M&A action.

### Policy implication

Given the role of the KPPU as the government agency responsible for maintaining fair business competition, we suggest that the KPPU should provide not only the list of the acquiring and acquired companies, but also more information such as the company’s profile, the financial reports (especially for companies that are not listed in IDX), and other information. Our results may be valuable for government decision-makers as part of the KPPU who need to support their decision-making processes based on financial reports. They need to look for the managerial ability to make strategic decisions to take M&A action.

### Limitations and ideas for future research

There are at least four limitations that should be considered in this research. First, this study did not consider the economic conditions in Indonesia, such as government policy changes and inflation. Future research may address this limitation by considering economic conditions, for example, the COVID pandemic situation that triggers more M&A actions. Second, this study did not consider the differences in culture between the acquirer and acquiree companies that might influence the company's performance after M&A. Since the consolidation of two or more companies involves different cultures, future research might address this aspect. The third limitation is related to the method of measuring managerial ability (MA). In this study, MA is measured using a ratio scale. Future research might consider using the nominal scale (dummy variable) with a high (more efficient) MA for 1 and a low MA for 0. Future research might also consider the dual role of the acquirer company's CEO, which may relate to the managerial ability necessary for M&A performance. The fourth limitation is related to the availability of M&A actions for nonpublic companies. This research only uses data for acquiring companies listed in IDX for operating performance related to market value. However, most of the M&As listed in the KPPU are related to nonpublic companies. It would be beneficial if KPPU also provided financial reports of M&A companies so that future research might study the effect of MA on the M&A of nonpublic companies.

## Data Availability

The raw data came from the published annual report and stock market information that was easily accessed from the Indonesia Stock Exchange (IDX).

## Abbreviations

MA: Managerial ability
M&A: Mergers and acquisitions
VRI: Value recognition index
MTBR: Market-to-book ratio
BHAR: Buy–hold abnormal return
KPPU: Business Competition Supervisory Commission
PER: Price to equity
Apindo: National Indonesian Employers Association
Bapindo: Indonesian Development Bank
JPFA: PT Japfa Comfeed Indonesia
DOC: Animal feed and commercial farm day-old chicken
PSAK: Indonesian Financial Accounting Standards
IDX: Indonesia Stock Exchange
IHSG: Composite stock price index
